# The effect of COVID-19 on the economy: Evidence from an early adopter of localized lockdowns

**DOI:** 10.7189/jogh.10.05002

**Published:** 2021-01-16

**Authors:** Kenzo Asahi, Eduardo A Undurraga, Rodrigo Valdés, Rodrigo Wagner

**Affiliations:** 1 Escuela de Gobierno, Pontificia Universidad Católica de Chile, Santiago, Región Metropolitana, Chile; 2 Centre for Sustainable Urban Development (CEDEUS), Chile; 3 Millennium Nucleus for the Study of the Life Course and Vulnerability (MLIV), Santiago, Chile; 4 Millennium Initiative for Collaborative Research in Bacterial Resistance (MICROB-R), Santiago, Chile; 5 Research Center for Integrated Disaster Risk Management (CIGIDEN), Santiago, Chile; 6 Business School, Universidad Adolfo Ibáñez, Santiago, Chile; 7 Growth Lab, Center for International Development, Harvard, Cambridge, Massachusetts, USA

## Abstract

**Background:**

Governments worldwide have implemented large-scale non-pharmaceutical interventions, such as social distancing or school closures, to prevent and control the growth of the COVID-19 pandemic. These strategies, implemented with varying stringency, have imposed substantial social and economic costs to society. As some countries begin to reopen and ease mobility restrictions, lockdowns in smaller geographic areas are increasingly considered an attractive policy intervention to mitigate societal costs while controlling epidemic growth. Nevertheless, there is a lack of empirical evidence to support these decisions.

**Methods:**

Drawing from a rich data set of localized lockdowns in Chile, we used econometric methods to measure the reduction in local economic activity from lockdowns when applied to smaller or larger geographical areas. We measured economic activity by tax collection at the municipality-level.

**Results:**

Our results show that lockdowns were associated with a 10%-15% drop in local economic activity, which is twice the reduction in local economic activity suffered by municipalities that were not under lockdown. A three-to-four-month lockdown had a similar effect on economic activity than a year of the 2009 great recession. We found costs are proportional to the population under lockdown, without differences when lockdowns were measured at the municipality or city-wide levels.

**Conclusions:**

Our findings suggest that localized lockdowns have a large effect on local economic activity, but these effects are proportional to the population under lockdown. Our results suggest that epidemiological criteria should guide decisions about the optimal size of lockdown areas since the proportional impact of lockdowns on the economy seems to be unchanged by scale.

Despite the historic approvals in the United Kingdom and the United States of a COVID-19 vaccine tested in a large clinical trial [1,2], non-pharmaceutical interventions are still the main strategies to control viral transmission in the COVID-19 pandemic [3-6]. These interventions range from individual-level recommendations, such as the use of facemasks or frequent hand-washing, to large-scale regulations and policies, such as large-scale lockdowns and non-essential business closures [7,8]. Several countries have achieved some control over the COVID-19 based on a combination of non-pharmaceutical interventions and high levels of testing and isolation of infected people [9-15]. However, several countries are going through the second wave of infections, and there is a substantial risk of a resurgence of the epidemic elsewhere [16-19]. Understanding these interventions' impacts is critical because they will most likely continue until an effective vaccine becomes available for a substantial proportion of the population [20]. There is still limited empirical evidence of the effects of interventions to prevent viral transmission [3,21]. Most intervention’s impact has been estimated using mathematical models [20,22-26]. The COVID-19 pandemic has already imposed an enormous global burden, with about 72 million cases and one million deaths reported so far, and substantial social and economic costs from epidemic control measures [27-33].

As countries have begun to reopen and ease mobility restrictions, localized lockdowns are increasingly considered a critical element of a non-pharmaceutical toolkit to control COVID-19 resurgence [9,10,23,34,35]. In contrast to nation-wide lockdowns, localized lockdowns are implemented over a limited geographical area, ranging from a neighbourhood to a city, including suburbs, districts, or towns. Localized lockdowns may be appealing to policy-makers because, in principle, they would allow countries to reopen and reclose specific jurisdictions based on local virus transmission dynamics. Large-scale lockdowns are unsustainable because of the high costs they impose on the population [15]. Thus, compared to large-scale interventions, localized lockdowns may control transmission hotspots while mitigating some social and economic costs. Policy-makers need to make decisions about COVID-19 control strategies, considering their epidemiological, social, and economic effects.

Epidemiological evidence is one of several criteria for decision-making regarding non-pharmaceutical interventions. For example, a policy-maker would want to understand if costs of foregone economic activity are proportional to the population under lockdown or whether per-person costs are mitigated or amplified when lockdowns are implemented at different administrative levels (eg, municipality, city, state, country). On the one hand, demand spill overs would suggest that people in a municipality could buy in stores of the neighbouring municipality and, through substitution, limit the economic fallout in the city as a whole. On the other hand, the fall in economic activity could be more than proportional if a lockdown affects supply chains, such as when workers cannot work in a neighbouring municipality because of mobility restrictions in their municipality of residence. The answer to this question is mostly empirical, as there are good arguments to both sides. However, there is limited and non-conclusive evidence on the economic costs of non-pharmaceutical interventions. Researchers in the United States have examined how non-pharmaceutical interventions have impacted unemployment insurance, employment, or store client traffic [28,30,36-40]. Some research suggests that lockdowns explain a small share of the total economic activity decline [28,36-38]. Others [30,39,40] have found that lockdowns play a relevant role in explaining the drop in economic activity. We test these effects in a setting where localized lockdowns were implemented intermittently at different administrative levels, thus allowing us to identify the impact of localized lockdowns on economic activity.

The World Health Organization declared South America as the new epicentre of COVID-19 on May 22, 2020 [41]. Despite implementing several epidemic control strategies early in the pandemic, including travel restrictions, school closures, and mandatory lockdowns, the pandemic has imposed a massive toll on the region. As of December 13, South America has reported more than 360 thousand deaths [42]. Adjusted by population, Argentina, Colombia, Chile, Brazil, and Peru are among the countries with the most reported COVID-19 infections and deaths globally. The epidemic is far from controlled [42-44]. While mostly failing to stop viral spread [43,45], Latin America is now facing the social and economic costs of large-scale non-pharmaceutical interventions.

Since the beginning of the epidemic, Chile has implemented localized lockdowns at the municipality level, the smallest administrative subdivision in the country, at various points in time (**Figure 1**). The government roughly defined the criteria for implementing localized lockdowns as a function of disease burden, growth in transmission, and health care capacity but did not define specific thresholds [46]. Epidemiological evidence suggests that localized lockdowns reduce epidemic growth [47], but their effectiveness is heavily affected by their duration, spill overs from neighbouring communities, and socioeconomic status of the population affected [48,49]. Localized lockdowns have helped contain the transmission of the virus in isolated areas. Still, they cannot control the epidemic in highly interdependent areas, such as municipalities within a metropolitan area [21].

**Figure 1:**
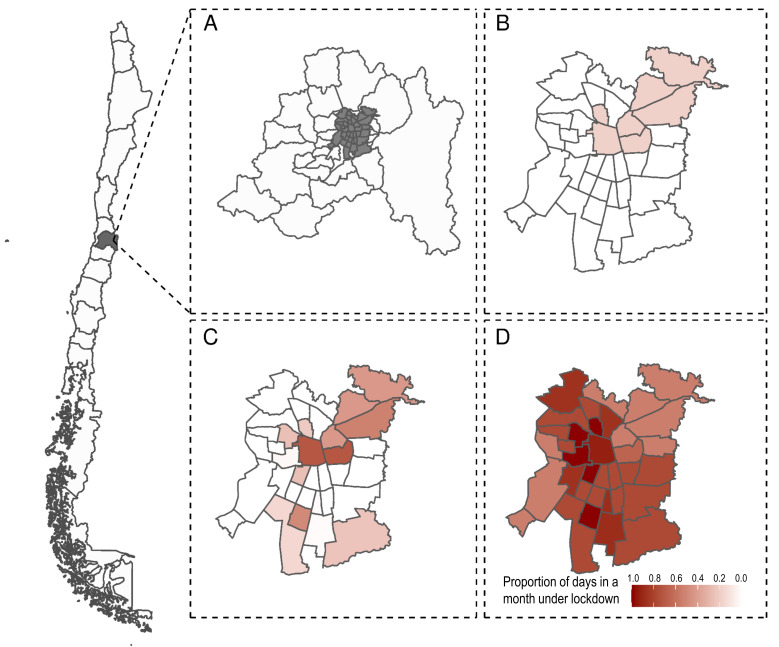
Illustration of localized lockdowns at the municipality level, Greater Santiago, Chile, March-May 2020. To control COVID-19 growth, the Ministry of Health implemented localized lockdowns at the country's municipality level, the smallest administrative subdivision. The figure illustrates these lockdowns implemented in Greater Santiago (A in grey) during different months: March (**B**), April (**C**), and May (**D**), where the colour scale represents the proportion of days in a month under lockdown for each municipality.

Drawing on a rich integrated data set, including value-added tax (VAT) revenues, population data, and daily incidence of laboratory-confirmed COVID-19 cases, we use econometric methods to empirically estimate the economic costs of these localized lockdowns in Chile. We hope these results will help inform policy implementation decisions in the context of the COVID-19 pandemic.

## METHODS

### Data

Value-added tax (VAT) applies to all goods with a flat rate of 19% in Chile. VAT is collected and paid monthly to the Chilean tax authority (Servicio de Impuestos Internos). Our data includes VAT at the municipality level, by all firms registered in the Chilean tax authority, for 2018-2020. VAT collection has a tight one-to-one relationship with GDP; it is, therefore, a good proxy for economic activity. Both variables cointegrate in time series and panel analysis; error correction models suggest that half-life deviations vanish in less than a year [50].

We used Chile’s 2017 National Census [51] to estimate each municipality's population and epidemiological surveillance records for COVID-19 from Chile’s Ministry of Health [46,52]. We obtained mobility data from the Data Science Institute at Universidad del Desarrollo [53,54]. Mobility data correspond to Chile’s largest telecommunications operator. Data on COVID-19, mobility, and population are publicly available on institutional websites [51-53]. The data on VAT used for this study are available from the corresponding author upon reasonable request and with permission of the Chilean tax authority.

### Analysis

We used the collection of the VAT as our dependent variable. Our lockdown variable corresponds to the proportion of days that a municipality *i* is in lockdown in a given month *t*:

Lockdown_i,t_ =  ∑ quarantine days_it_/Total month’s days_t_

**Figure 2:**
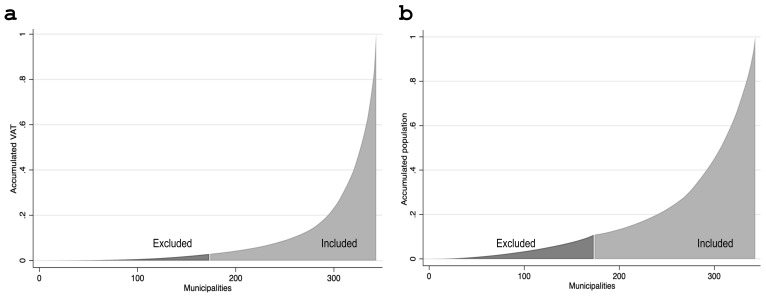
VAT and population cumulative distribution across all municipalities. Panel (a) shows the proportion of total 2018 VAT considered in our baseline sample. We sorted the 343 municipalities in our data set in ascending order by 2018 VAT. We calculated the accumulated tax from the one with the lowest to the highest VAT level. Municipalities not considered in our baseline sample account for 2.9% of the total 2018 VAT (darker area), while the remaining 97.1% (lighter area) is in our preferred sample. Panel (b) shows the proportion of the total population, according to the 2017 Census within our preferred sample. In this case, we sorted the municipalities in ascending order. We then calculated the total population's accumulated percentage not considered in our sample, which is 10.9% (darker area). Hence, the remaining 89.1% (lighter area) is in our sample.

We limited our analysis to the 170 municipalities with above-median total VAT in 2018, excluding mostly small and rural municipalities. This preferred sample of municipalities includes 97% of Chile’s 2018 VAT and 89% of the population (**Figure 2**). Our sample also excluded the three municipalities that concentrate large-company headquarters (Santiago, Las Condes, and Providencia), such as banks and mining companies, because VAT data in these municipalities do not reflect local economic activity.

Our main empirical specification is a two-way fixed-effects model:

Δ%VAT_it_ = β + β_1_lockdown_it_ + β_2_X_it_ + γ_i_ + δ_t_ + ε_it_

where *∆%VAT_it_* corresponds to the percent variation of total VAT collected in municipality *i* at month *t* in 2020 relative to the same month in 2019. *lockdown_it_* is our variable of interest and represents the proportion of days in a month that a municipality was under lockdown. γ_i_ and δ_t_ correspond to municipality and time fixed-effects, respectively. A distinctive feature of our setting is that *lockdown_it_* effectively changes by municipality and month, providing a variation that allows for a plausible estimate of effects (**Figure 1**). We controlled for threat or risk perception [55] and social distance by adding new monthly COVID-19 cases or new monthly COVID-19 deaths in the municipality *i* at time *t* (variable X_it_) as covariates. For instance, people may not open their businesses or spend in the local economy because they fear COVID-19 contagion, independent of whether their municipality is under lockdown or not.

Similar to virus transmission spill overs, the economic effects of localized lockdowns within a city or in a conurbation may differ from more relatively isolated municipalities with no neighbouring urban areas (“standalone” municipalities). To examine whether the impact of lockdowns on economic activity is heterogeneous depending on whether municipalities belong to a conurbation or are a standalone municipality, we used the following regression specification:

Δ%VAT_it_ = β_0_ + β_1_lockdown_it_ + β_2_standalone_i_ + β_3_standalone_i_ × lockdown_it_  + γ_i_ + δ_t_ + ε_it_

where *standalone_i_* takes a value of one for standalone municipalities and zero otherwise.

The economic effects of localized lockdowns may differ depending on the area under lockdown – for example, at the municipality or conurbation level. To examine this question, we also ran our analysis comparing all municipalities within a conurbation with standalone municipalities. We weighted the number of days in lockdown in month *t* of each municipality *i* belonging to the conurbation *c* according to the total 2018 VAT:


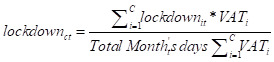


We estimated deaths and COVID-19 cases as a weighted average of deaths in municipalities within the conurbation, using the municipality’s population as the weight. Hence, the equation describing per capita COVID deaths in each conurbation is as follows:


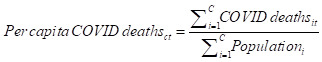


Last, we investigate how mobility at the municipality-level affects economic activity. We used a mobility index based on cell phone data. The index was calculated from anonymized aggregate records of mobile telephones in Santiago, which describe trips within and between municipalities. Data are not based on the mobile phone's exact location but on the antenna to which the phones were connected. Each trip is defined by the person’s mobile phone moving between antennas [53,54].

## RESULTS

### Descriptive statistics

**Figure 3:**
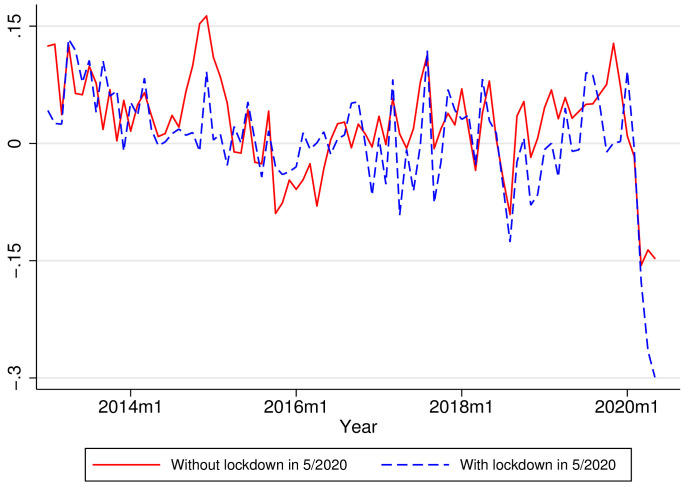
Median of the real value-added-tax (VAT) year-on-year growth rates. The graphs show the median of VAT growth rates for municipalities under lockdown in May 2020 (blue) and municipalities that were not under lockdown (red). The median of the value-added-tax (VAT) growth rate in May 2020 for municipalities with and without lockdown is 2.67 and 5.37 standard deviations lower than the mean of such medians in the 2006-2019 period. The sample of municipalities includes municipalities over the 50^th^ percentile of the total 2018 VAT.

**Figure 4:**
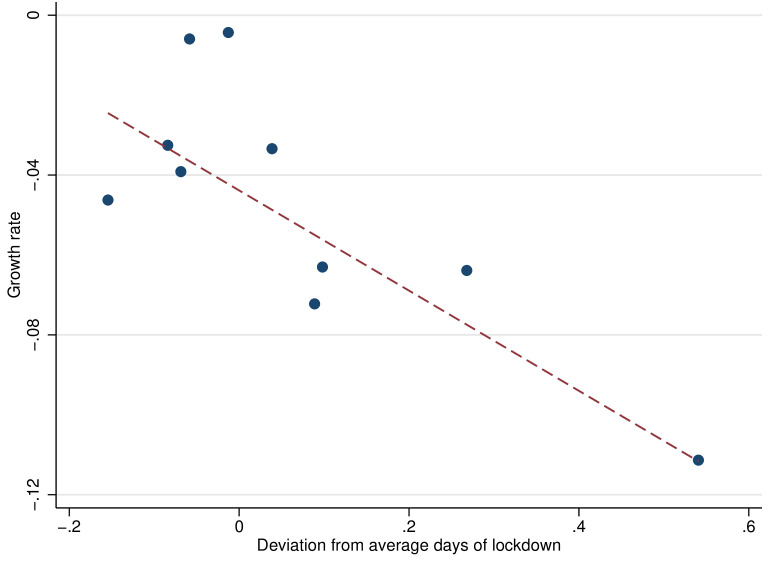
Effect of lockdown on value-added-tax (VAT) collection for January 2020 through May 2020, controlling for month and municipality fixed effects. The results show the association between lockdown on VAT collection for January 2020 through May 2020, controlling for month and municipality fixed effects. We group the municipalities of our baseline sample into equal-sized bins according to days of lockdown between January 2020 and May 2020. Each dot represents the mean VAT collection growth rate (y-axis) and the mean deviation from lockdown as a percentage of a month (x-axis) within each bin. Each bin has 17 municipalities. The red dashed line represents the population regression line.

**Table 1** shows the main descriptive statistics of our sample. **Figure 3** shows the longitudinal effects of lockdown. As a benchmark, municipalities without a localized lockdown saw a 15% drop in VAT collection in April-May 2020 compared to the same months of 2019. By contrast, municipalities with lockdown suffered a more substantial decline of 25%-30% in VAT collection, again measured vis-à-vis the previous year. **Figure 4** shows a cross-section, considering month and municipality fixed-effects. The figure shows a clear relationship between the extent of lockdowns and the decline in VAT.

**Table 1:** Descriptive statistics related to localized lockdowns in Chilean municipalities, March-May 2020.

	N	Municipalities	Mean	SD	
**VAT log growth rate periods, compared to the same month in the previous year:**				
Before the outbreak: January-February 2020	340	170	0.083	0.423	
During the pandemic: March-May 2020	510	170	-0.139	0.319	
**Lockdown (% of days):**				
March-May 2020	510	170	0.082	0.227	
Conditional on one day at least	153	51	0.273	0.348	
**Mobility index (mobile phones):**				
Before COVID-19 (March 1-15)	170	170	8.03	3.24	
During COVID-19 (March 16-May 31)	510	170	6.12	2.57	
**New COVID-19 deaths per million population:**				
March 2020	170	170	2.13	6.61	
April 2020	170	170	20.32	27.90	
May 2020	170	170	108.95	147.54	
**New COVID-19 incidence per million population:**				
March 2020	168	168	59.54	119.57	
April 2020	170	170	633.38	729.48	
May 2020	170	170	3160.5	4319.0	

SD – standard deviation, VAT – value-added tax

*The VAT year-over-year growth rate is calculated as the difference in logarithms of VAT for each month and each municipality relative to 12 months before. As such, a number like 0.08 is approximately an 8% drop vis-à-vis the previous year. In the VAT growth rate for “March-May 2020,” we include the growth rate for each month-municipality in that period. We consider that the disruption of the COVID-19 pandemic on mobility occurred on March 16 because the government closed all schools on that date [46].

### Multivariate analysis

#### Municipality level

**Table 2** presents our baseline results for the effect of lockdowns on economic activity. **Table 2**, column (1) shows that one month of lockdown decreases monthly VAT around 12.5% (β = -0.125; 95% confidence interval (CI) = -0.220, -0.031; *P* = 0.009). The coefficient or the effect of lockdowns has about the same magnitude when restricting the sample to municipalities with at least 50% of the urban population (**Table 2**, column 2; β = -0.132; 95% CI = -0.228, -0.035; *P* = 0.008). **Table 2**, column (3) shows the results for municipalities with less than 50% of rural population and excluding observations from Greater Santiago. To assess our estimates' robustness, we excluded municipalities in Greater Santiago, the conurbation in Chile with the highest proportion of municipalities in lockdown between March and May 2020. We found that VAT decreases 16 percentage points for each month of lockdown, but the coefficient is only significant at the 90% level (β = -0.162; 95% CI = -0.350, 0.0268; *P* = 0.093).

**Table 2:** Regressions results for the effect of one month localized lockdown on total VAT collection, estimated with two-way fixed effects at the municipality level, January-May 2020

Dependent variable: VAT growth	Baseline	Excluding rural units	Excluding Greater Santiago	Conurbations	Conurbations excluding Greater Santiago	Conurbations and standalone municipalities	As in (1) controlling for deaths	As in (1) controlling for cases
	**(1)**	**(2)**	**(3)**	**(4)**	**(5)**	**(6)**	**(7)**	**(8)**
Lockdown	-0.125§	-0.132§	-0.162†	-0.161‡	-0.153	-0.230§	-0.125†	-0.135§
	(0.048)	(0.049)	(0.096)	(0.064)	(0.130)	(0.059)	(0.071)	(0.052)
Standalone × lockdown						-0.059		
						(0.104)		
Standalone						-0.005		
						(0.038)		
New deaths per 100 000							0.00004	
							(0.002)	
New log cases per 100 000								0.002
								(0.005)
Observations	850	785	570	360	195	455	850	850
Adjusted R^2^	0.352	0.369	0.356	0.352	0.278	0.171	0.351	0.351
Time effect	YES	YES	YES	YES	YES	YES	YES	YES
Municipalities	170	157	114	72	39	91	170	170

VAT – value-added tax

*All specifications have both geography and time fixed effects. Robust standard errors in parenthesis. Column (1) shows regression results for the baseline sample (ie, municipalities over the 50^th^ percentile of total 2018 VAT). Column (2) excludes units, with over fifty percent of the rural population. Column (3) is the same as (2) but excluding municipalities in Greater Santiago, the capital. Columns (4) includes only municipalities that are part of a large conurbation. Column (5) is the same as column (4), excluding municipalities in Greater Santiago. Column (6) includes municipalities that are part of large conurbations and standalone municipalities (Angol, Antofagasta, Arica, Aysén, Calama, Castro, Chañaral, Colina, Copiapó, Curicó, Osorno, Ovalle, Puerto Montt, Puerto Natales, Punta Arenas, Valdivia, and Vallenar). Columns (7) and (8) consider the baseline sample and controls for contemporaneous COVID-19 new deaths and lagged incidence per 100 000 population, respectively, at the municipality level.

†*P* < 0.10.

‡*P* < 0.05.

§*P* < 0.01.

We then limited our sample to urban municipalities (n = 72) that are part of a conurbation (**Table 2**, column 4). One month of lockdown results in a monthly VAT decrease of 16 percentage points (β = -0.161; 95% CI = -0.287, -0.034; *P* = 0.013). We found similar results when excluding Greater Santiago (**Table 2**, column 5; β = -0.153; 95% CI = -0.410, 0.103; *P* = 0.240).

We added an interaction term to examine whether lockdowns had a different effect on VAT in municipalities that are part of a conurbation or in standalone municipalities. The results in **Table 2**, column (6) show a 23% decline in monthly VAT collection due to a one-month lockdown (β = -0.230; 95% CI = -0.345, -0.115; *P* < 0.001). However, we did not find evidence of a differential effect for standalone municipalities relative to municipalities in conurbations.

Last, we examined whether perceived threat or risk from new COVID-19 deaths or new cases could be an omitted variable bias in the effect of local lockdowns on economic activity. **Table 2**, Column (7) includes the municipality’s one-month per-capita COVID deaths per 100 000 population as control. The lockdown effect is roughly the same as in column 1 (β = -0.125; 95% CI = -0.265, 0.013; *P* = 0.077). Controlling for COVID-19 monthly incidence per 100 000 population **Table 2**, Column (8) shows that one month of lockdown results in a thirteen percent decrease in VAT collection (β = -0.135; 95% CI = -0.237, -0.033; *P* = 0.010). Results are robust to using one-month lagged COVID-19 deaths and cases.

Overall, **Table 2** suggests that one month of lockdown would reduce economic activity by 10%-15%, robust to several model specifications. Notably, the effect size is not affected when controlling for COVID-19 deaths or case incidence, suggesting that this sample's lockdown effect goes over and above the impact of perceived threat or risk of contagion.

#### Conurbations and standalone municipalities

Next, we examined the effects of lockdowns on VAT when analysed for conurbations or standalone municipalities (**Table 3**). The objective was to test whether the effects of lockdowns were different when there were no spill overs from closely interdependent neighbouring areas. For the analysis, we collapsed municipalities into conurbations. Our sample now had eighteen conurbations and seventeen standalone municipalities in our baseline sample.

**Table 3:** Regressions results for the effect of one month localized lockdown on total VAT collection, estimated with two-way fixed effects for conurbations and standalone municipalities, January-May 2020

Dependent variable: VAT growth	All Conurbations and standalone municipalities	Excluding Greater Santiago	As in (1) interacting lockdown & standalone	As in (1) with per capita deaths	As in (1) with log per capita incidence
	**(1)**	**(2)**	**(3)**	**(4)**	**(5)**
Lockdown	-0.184‡	-0.188†	-0.243§	-0.126	-0.157
	(0.089)	(0.020)	(0.064)	(0.106)	(0.116)
Standalone			-0.00294		
			(0.044)		
Standalone × lockdown			-0.042		
			(0.105)		
New deaths per 100 000				-0.007	
				(0.006)	
New log cases per 100 000					-0.014
					(0.027)
Observations	175	170	175	175	175
Adjusted R^2^	0.325	0.322	0.101	0.326	0.323
Units	35	35	35	35	35
Conurbations	18	18	18	18	18
Standalone municipalities	17	17	17	17	17

VAT – value-added tax

*All specifications have both geography (conurbation, standalone municipalities) and time fixed-effects. Robust standard errors in parenthesis. Localized lockdowns at the conurbation level are aggregated and weighted by total 2018 VAT. COVID-19 deaths and cases are calculated at the conurbation level.

†*P* < 0.10.

‡*P* < 0.05.

§*P* < 0.01.

**Table 3**, column (1), shows a statistically significant decline in monthly VAT collection of around 18% (β = -0.184; 95% CI = -0.360, -0.009; *P* = 0.042). Because Greater Santiago had the largest number of municipalities with lockdown, we dropped Greater Santiago from the sample to test our results (**Table 3**, column 2). The effect's magnitude remained but was significant only at the 90% level (β = -0.188; 95% CI = -0.382, 0.051; *P* = 0.056). In **Table 3**, column 3, we examined whether there was a differential effect for standalone municipalities. The results show that one month of lockdown results in a significant decrease of 24% of VAT collection (β = -0.243; 95% CI = -0.370, -0.117; *P* < 0.001). We did not find evidence for a differential effect in standalone municipalities. However, the coefficient in **Table 3****,** column 3, was not statistically different from the coefficient in **Table 3**, columns (1) and (2).

Last, we examined whether the lockdown effect was different from the perceived threat or risk from COVID-19. In **Table 3**, columns (4) and (5) show lockdowns were no longer statistically significant at conventional levels (*P* = 0.240 and *P* = 0.175, respectively). However, the coefficient’s sign was still negative and about the same magnitude as the coefficient in **Table 3**, columns (7) and (8). The joint significance test for the proportion of the month under lockdown and lagged per capita COVID-19 deaths and incidence was significant (F = 3.84, *P* < 0.05; F = 2.81, *P* = 0.064, respectively). Thus, working with data at the conurbation-level instead of the municipality-level makes it harder to disentangle the effect of lockdowns. This difficulty is partly explained by insufficient statistical power and by limited variation in the lockdown variable. The last columns of Table 3 reinforce the advantage of our baseline setting at the municipal level, with more sizable variation in the lockdown (key) variable.

Table S1 in the **Online Supplementary Document** also shows that our baseline results are robust to controlling for a measure of cell phone-based mobility. However, we also argue that it might be misleading to control for mobility since it is one of the main mediating channels by which lockdown affects economic activity (see Appendix S1 in the **Online Supplementary Document** for further discussion).

## DISCUSSION

Our results suggest that a full-month lockdown explains a drop in activity of the order of 10-15 percentage points, almost twice the reduction for non-locked down areas. While the expected sign of the effect of lockdowns on economic activity might be obvious, its magnitude is not.

These estimates are large. Our estimates suggest that a three-to-four-month lockdown would reduce economic activity by approximately the same amount that the recession affected the Chilean economy in the (whole) year 2009. During the 2009 Great Recession, GDP declined by 1.1% instead of growing by 3.7% [56]. These three to four months only consider the additional effect of lockdowns. If one considered the whole drop in economic activity, the magnitude would be twice as much (in two months under lockdown in 2020, the GDP decline is comparable to the annual decrease in 2009).

Another way of thinking quantitatively about the magnitude and implications of our baseline estimate is in terms of employment. Assuming a standard short-run labour-to-economic activity elasticity of around 0.3-0.5, as suggested by an OECD study [40], a one-month lockdown would imply a drop of about 6% in monthly employment. We estimate this illustrative 6% fall in monthly employment by multiplying the coefficient of -0.15 in **Table 2** by an average short-run labour-to-economic activity elasticity of 0.4.

It is also useful to contrast our results with the polar case of South Korea, without lockdowns. Aum et al. found that a one-per-thousand increase in the infection rate was associated with an employment loss of 2 to 3 percent. Extrapolating this result to the United States and the United Kingdom, which had large-scale lockdowns, Aum et al. [30] argue that only half of the 5 to 6 percent drop in job losses in these Western economies might be attributable to lockdowns. The rest would be from social panic, some other large-scale non-pharmaceutical intervention, such as school closures, or demand effects. This similar effect of areas with and without lockdown seems consistent with our findings. Importantly, we obtained our results from a direct test in the same sample, instead of extrapolating across countries. The relatively large effect of lockdowns has not yet been found empirically in the United States. For instance, Bartik et al. found that the relative impact of lockdowns was smaller, explaining 1/6 of the total fall during the COVID-19 pandemic. Our results show that lockdowns explain half of the effect, both in the raw time series (**Figure 3**) and in the main regressions (**Table 2**). Thus, we offer a qualification to Brzezinksi et al. [57], who found that not imposing lockdowns barely improves economic performance while drastically increasing medical costs. This baseline drop probably includes threat or risk perception and includes other economic channels, like lower spending [31].

Epidemiological evidence suggests that localized lockdowns reduce epidemic growth [47], but their effectiveness is affected by spill overs from neighbouring areas where there is economic interdependence, such as in a city [21]. From an epidemiological standpoint, governments may desire to implement localized lockdowns at the city-level, where “buffer” zones exist to minimize transmission networks [34]. We examined localized lockdowns at different scales to understand their relative economic costs, understanding that this is only one portion of the relevant cost-benefit calculation. Our findings suggest no disproportionate economic gains from unlocking a part of the city. Our estimated effects of lockdowns on the economy are unchanged by scale. The plausible channels that mitigate or amplify the economic impact in the case of a widespread vis-à-vis a local lockdown do not seem critical, at least in our study setting, suggesting the convenience of implementing localized lockdowns at the city or commuting area levels, if epidemiologically appropriate.

Economic problems could also feedback into health through several channels. For instance, a drop in economic activity of 10-15 percentage points is relevant because lockdowns can affect government budgets, even in the long term. For example, Frenier et al. [58] argue that several states in the USA will probably face severe budget deficits from tax revenue reductions from the pandemic. Further, an economic downturn may prompt many deaths of despair and mental illness from unemployment and isolation [59].

Our estimates have limitations. First, we used a tax payment as a proxy for economic activity. Nonetheless, we also have VAT and survey-based employment at the regional level in Chile. We found a statistically significant elasticity of 0.3 between the drop in VAT and the decline in total employment (including self-employed), consistent with short-run output-employment elasticities in the literature (34). Another limitation is that informal economic activity is, by its very nature, not directly captured in our measures of VAT. However, compared to Latin America (53%), Chile has relatively low levels of informality (30%) [60], which have declined in the past decade [61]. Still, many households depend on daily wages from informal employment, which has critically affected their capacity to comply with some non-pharmaceutical interventions such as localized lockdowns [48,49]. The World Bank has recently underscored the pandemic's adverse effects on informal employment and businesses, which are harder to reach through policy instruments such as subsidies or payment deferrals [27]. We lack data on informal activity during the pandemic. However, we have some indirect evidence of the pandemic's large economic effects on informal activity through data from informal settlements in Chile. A nationally representative panel survey of informal settlements showed that about 75% of individuals had lost more than half their income since the pandemic began [62], a substantially larger decline in income compared to the general population. These data suggest that our estimates of the effects of COVID-19 on the Chilean economy may be conservative.

Our study may also have other confounders. For instance, the government gave some leeway on when to pay taxes, and we could only examine monthly-level observations. Nevertheless, there are no apparent reasons why these confounders may interact with lockdowns. These confounders may have also introduced measurement error in our tax measures. This measurement error would have increased our standard errors, making it more difficult to get statistical significance. Nevertheless, we did get relevant and robust estimates across various specifications, which mitigates these concerns.

## CONCLUSIONS

We used a rich data set of localized lockdowns in Chile to measure their effect on economic activity. We find sizeable impacts of lockdowns, doubling the drop in economic activity compared to non-treated municipalities, and robust to several model specifications and controls. As many countries are beginning to reopen and ease mobility restrictions, localized lockdowns can be a critical tool to control COVID-19 resurgence while minimizing economic impact. We found no evidence that localized lockdowns generate a proportionally larger or smaller effect in the economy when applied to areas of different sizes. Critically, our results suggest that epidemiological criteria should guide decisions about the optimal size of lockdown areas since the proportional effects of lockdowns on the economy seem to be unchanged by the geographic scale of the restrictions.

## Additional material

Online Supplementary Document
