# Supplementary material for: The effect of COVID-19 on the economy: Evidence from an early adopter of localized lockdowns
Source: J Glob Health. 2021 Jan 16;11:05002. doi: 10.7189/jogh.10.05002 (PMC7897430; doi:10.7189/jogh.10.05002)
Supplement: Online Supplementary Document [file jogh-11-05002-s001.pdf]

Journal of Global Health

Online Supplementary Document

The effect of COVID-19 on the economy: evidence from an early adopter of localized lockdowns

Kenzo Asahi PhD[1,2], Eduardo A. Undurraga PhD[1,3,4,5]*, Rodrigo Valdés PhD[1,5], Rodrigo Wagner PhD[6,7]

[1]*Escuela de Gobierno, Pontificia Universidad Católica de Chile, Santiago, Región Metropolitana, Chile.*

[2]*Centre for Sustainable Urban Development (CEDEUS), Chile*

[3] *Millennium Nucleus for the Study of the Life Course and Vulnerability (MLIV), Santiago, Chile*

[4] *Millennium Initiative for Collaborative Research in Bacterial Resistance (MICROB-R), Santiago, Chile*

[5]*Research Center for Integrated Disaster Risk Management (CIGIDEN), Santiago, Chile*

[6]*Universidad Adolfo Ibáñez, Santiago, Chile*

[7]*Growth Lab, Center for International Development, Harvard, Cambridge, USA*

***Address for correspondence**: Eduardo A. Undurraga, Escuela de Gobierno, Pontificia Universidad Católica de Chile. Vicuña Mackenna 4860, Macul CP 7820436, Región Metropolitana, Chile; email: eundurra@uc.cl; phone: +56 (2) 2354 9536. ORCID: 0000-0002-4425-1253

Emails: kasahi@uc.cl, eundurra@uc.cl, rodrigo.valdes@uc.cl, rodrigo.wagner@uai.cl

Appendix Table S1. Regressions results for the effects of mobility and localized lockdown on VAT growth using ordinary least squares regressions and two-stage least squares fixed effects estimates, January to May 2020*

**Panel A: Two stages least squares**	(1)	(2)	(3)
Dependent variable: VAT growth	OLS 2WFE	OLS 2WFE	IV 2WFE
Lockdown as a percentage of a month	-0.131‡		
	(0.053)		
Log mobility	-0.036	0.043	0.791‡
	(0.140)	(0.130)	(0.032)
Observations	850	850	850
Adjusted R^2	0.351	0.349	
Municipalities	170	170	170
**Panel B: First stage**			
Dependent variable: Log Mobility	(1)	(2)	(3)
Lockdown as a percentage of a month			-0.159§
			(-5.83)
Observations			850
Adjusted R^2			0.970
Municipalities			170

OLS – Ordinary least squares, VAT – value-added tax, 2WFE – two-way fixed effects

*Robust standard errors in parenthesis. Panel A shows the impact of mobility and lockdown on VAT growth using OLS, and two-stage least squares fixed effects estimates for the baseline sample. We consider a mobility index as an endogenous variable for January to May that reacts to lockdowns. For each municipality in January and February, we imputed the mean of the daily mobility index during March's first fifteen days. Column (1) controls for mobility using the logarithm of the daily average of the mobility index at the municipality level. Column (2) uses the mobility index to explain variations in VAT with OLS 2WFE. In column (3), we use lockdown as an instrumental variable for mobility.

†P<0.10, ‡P<0.05, §P<0.01.

Mobility

We investigated whether mobility affects economic activity (**Table S1**). Mobility data were estimated based on a mobility index, calculated from anonymized aggregate records of mobile telephones in Santiago, which includes trips within and between municipalities. In a simple regression, with and without controlling for lockdowns, the mobility index had a non-significant effect on economic activity. **Table S1**, column (1) suggests that lockdowns continue having sizable effects even after controlling for mobility. We found no significant effects of mobility on economic activity (**Table S1**, column 2).

Since lockdowns and mobility could work in the same mechanism, in column (3) of **Table S1**, we use the method of instrumental variables. Instrumental variables are a way to analyze how lockdown-induced shocks to mobility impact economic activity. For expositional purposes, this is done in two stages. In the bottom panel of column (3), the so-called first-stage has a good fit, meaning that lockdowns impact mobility. On the top panel, the second stage regresses VAT on the lockdown-induced mobility changes, which were calculated in the first stage above. This second stage has a large and significant coefficient of 0.79. These two stages are jointly estimated to estimate standard errors correctly.

Importantly, this method of instrumental variables tries to decompose the effect of lockdowns on mobility and the subsequent impact of mobility on economic activity. The first coefficient means that a month of lockdown changes monthly mobility by minus 15%. The second coefficient means that lockdown-induced mobility changes VAT by +79%. The multiplication of these effects gives a sense of the net impact of lockdowns on VAT. The multiplication (-0.15×0.79) yields a minus 0.11. This result is reassuring because it falls within the range of our baseline estimates in **Table 2**. Notably, while there could be transmission mechanisms by which lockdowns affect economic activity beyond mobility, these results suggest that mobility is the leading mechanism.
